# Yield prediction in a peanut breeding program using remote sensing data and machine learning algorithms

**DOI:** 10.3389/fpls.2024.1339864

**Published:** 2024-02-20

**Authors:** N. Ace Pugh, Andrew Young, Manisha Ojha, Yves Emendack, Jacobo Sanchez, Zhanguo Xin, Naveen Puppala

**Affiliations:** ^1^United States Department of Agriculture, Crop Stress Research Laboratory, Lubbock, TX, United States; ^2^Agricultural Science Center at Clovis, New Mexico State University, Clovis, NM, United States

**Keywords:** artificial intelligence, crop yield, growth curves, machine learning, peanut, plant breeding, remote sensing, unmanned aerial vehicle

## Abstract

Peanut is a critical food crop worldwide, and the development of high-throughput phenotyping techniques is essential for enhancing the crop’s genetic gain rate. Given the obvious challenges of directly estimating peanut yields through remote sensing, an approach that utilizes above-ground phenotypes to estimate underground yield is necessary. To that end, this study leveraged unmanned aerial vehicles (UAVs) for high-throughput phenotyping of surface traits in peanut. Using a diverse set of peanut germplasm planted in 2021 and 2022, UAV flight missions were repeatedly conducted to capture image data that were used to construct high-resolution multitemporal sigmoidal growth curves based on apparent characteristics, such as canopy cover and canopy height. Latent phenotypes extracted from these growth curves and their first derivatives informed the development of advanced machine learning models, specifically random forest and eXtreme Gradient Boosting (XGBoost), to estimate yield in the peanut plots. The random forest model exhibited exceptional predictive accuracy (R^2 ^= 0.93), while XGBoost was also reasonably effective (R^2 ^= 0.88). When using confusion matrices to evaluate the classification abilities of each model, the two models proved valuable in a breeding pipeline, particularly for filtering out underperforming genotypes. In addition, the random forest model excelled in identifying top-performing material while minimizing Type I and Type II errors. Overall, these findings underscore the potential of machine learning models, especially random forests and XGBoost, in predicting peanut yield and improving the efficiency of peanut breeding programs.

## 1 Introduction

Peanuts (*Arachis hypogaea*) are a legume crop of immense economic and nutritional importance worldwide (Fletcher and Shi, 2016; Variath and Janila, 2017). They are a crucial component of the agri-food system, contributing significantly to global food security due to their rich nutritional composition (Valentine, 2016). Primarily, peanuts are renowned for their high protein content, providing about 25.8 grams of protein per 100 grams, which contributes substantially to dietary protein intake (Davis and Dean, 2016). Additionally, they are a rich source of healthy fats, particularly monounsaturated fats, which are associated with cardiovascular health benefits (Kris-Etherton et al., 1999; Settaluri et al., 2012). Peanut agroecosystems have been shown to likely increase the below ground metabolic activity in semi-arid systems (Laza et al., 2023) and show high levels of physiological adjustments under elevated CO_2_ conditions, which may increase drought resilience in future climates (Laza et al., 2021). Despite the substantial value of peanuts and their significance in global food security, there are considerable challenges associated with their genetic improvement. Although reliable, traditional peanut breeding methods are time-intensive processes that often require multiple selection cycles to develop improved cultivars (Chamberlin, 2019). This slow rate of progress in peanut breeding is a hurdle for the industry, particularly in the face of evolving challenges such as changing climate conditions and emerging pests and diseases (Faye et al., 2018; Obasa and Haynes, 2022; Haerani et al., 2023; Puppala et al., 2023). Peanuts have seen great improvements over the past decades; however, as is the case in other crop species, phenotyping remains a considerable bottleneck in peanut breeding pipelines and a chief limiting factor for overall rates of genetic gain in the crop (Furbank and Tester, 2011; Yang et al., 2020).

It is particularly difficult to directly estimate yield in peanuts using proximal and remote sensing methodologies. This difficulty stems from the unique growth characteristic of the peanut plant, wherein the gynophores, or “pegs,” grow downward into the soil after pollination (Boote, 1982; Moctezuma and Feldman, 1999). This subterranean growth, while advantageous for the plant in terms of accessing vital soil nutrients, makes non-invasive yield estimation incredibly challenging. Traditional methods for estimating yield typically require destructive sampling, which may not be efficient or desirable in many cases (Marshall and Thenkabail, 2015). Thus, developing feasible, non-destructive methods to model and predict potential yield in peanuts is a pressing need in the context of modernizing and streamlining peanut improvement systems.

In recent years, high-throughput phenotyping (HTP) via remote and proximal sensing has emerged as an important field for modern plant breeding, enabling crop improvement scientists to screen vast populations of plants rapidly and efficiently (Huang et al., 2019; Virnodkar et al., 2020; Tao et al., 2022). Traditional field-based phenotyping methods often involve manual measurements, which can be time-consuming, expensive, labor-intensive, and subject to human error (Pauli et al., 2016). Remote sensing, in contrast, utilizes state-of-the-art imaging technology to collect plant data in a non-destructive and automated manner, significantly reducing the time and cost associated with phenotypic data collection (Lobos et al., 2017; Araus et al., 2018; Janni and Pieruschka, 2022). Unmanned Aerial Vehicles (UAVs), or “drones” equipped with Red-Green-Blue (RGB) and multispectral cameras, can capture a wide array of data at both the individual plant and crop canopy levels (Thorp et al., 2018). Red-Green-Blue imagery can be processed using structure-from-motion (SfM) to estimate structural traits such as plant height and canopy width or leaf area index (Shi et al., 2016; Qi et al., 2020; Sarkar et al., 2020). The data obtained from remote sensing technologies can also be processed to derive a plethora of vegetation indices, which serve as critical tools in crop phenotyping and stress detection. Vegetation indices such as Excess Green Index (EG or ExG) and Normalized Difference Vegetation Index (NDVI) are often used to assess plant vigor, photosynthetic activity, and stress responses in a variety of crops, including peanuts (Rouse et al., 1974; Nijland et al., 2014; Zerbato et al., 2016). Perhaps most importantly, UAVs and other remote sensing technologies allow for efficient, repeated collection of data for the traits they measure, allowing researchers to produce multitemporal growth curves.

Multitemporal growth curves are derived from repeated, high-resolution remote sensing measurements taken throughout the crop growing season, enabling researchers to track the temporal progression of plant development and health with detail (Pugh et al., 2018; Bustos-Korts et al., 2019; Shammi and Meng, 2021). The high temporal resolution of repeated measurements allows the capture of physiological transitions such as the onset of flowering or maturity, which are critical growth stages that strongly correlate with final yield (Awal and Ikeda, 2003; Carneiro et al., 2019). The successful application of growth curves in yield modeling and prediction has been demonstrated in a variety of crops. For example, multitemporal measurements have been used to establish growth curves in tomatoes, employing these curves to predict yield prior to harvest (Chang et al., 2021). In Ashapure et al. (2020), multitemporal UAV data were used to develop a yield estimation framework in cotton. The successful application of this approach in these and other crops suggests a promising future for the application of similar methodologies in peanut yield prediction. The immense power of these growth curves lies in their ability to reveal latent phenotypes, which refer to traits or characteristics that are not directly observable but can be derived or inferred from the data (Ubbens et al., 2020; Lane and Murray, 2021). This concept stems from the premise that plant development is a dynamic process influenced by a multitude of factors, many of which may not be readily apparent in a singular, static snapshot of a crop field. Latent phenotypes could include characteristics derived from growth-rate trends, onset of key phenological stages, response to environmental stressors, and countless others (Gage et al., 2019; Feldmann et al., 2021). Once latent phenotypes are extracted, artificial intelligence (AI) can be used to predict traits of interest in peanuts, such as yield.

The integration of Artificial Intelligence (AI) in crop improvement programs has shown promising advancements in recent years. Machine learning (ML) models, such as Random Forest (RF) and eXtreme Gradient Boosting (XGBoost), have emerged as powerful tools in predicting various crop attributes (Thilakarathne et al., 2022; Zheng et al., 2022; Nazari et al., 2023). Both models are decision tree-based models that are commonly applied toward regression tasks, particularly for the prediction or modeling of key quantitative traits. Random Forest is an ensemble method that uses multiple decision trees during the training process where each “tree” makes its own prediction, and the final output is usually the mean of these predicted values (Breiman, 2001; Belgiu and Drăguţ, 2016). EXtreme Gradient Boosting is an advanced implementation of gradient boosting algorithms that builds trees sequentially, so that each new tree can correct the errors made by each previous tree, resulting in reduced error (Chen and Guestrin, 2016). Recent research in peanuts has shown that ML models can potentially be used to estimate various important traits via the use of the values of visual bands in RGB and multispectral imagery (Bagherian et al., 2023; Shahi et al., 2023). However, AI powered by remote sensing data has not yet been used across multiple years and a diverse set of material to assess models for their robustness in peanut (Bagherian et al., 2023; Shahi et al., 2023). Therefore, it is imperative that ML models to predict yield in peanut are built that are highly robust and remain applicable to unseen data; that is, data that have not yet been encountered by the models (Yoosefzadeh Najafabadi et al., 2023). To that end, the inclusion of multiple, varied environments and a population that has a large degree of heterogeneity is critical to ensure that ML models perform acceptably in this role.

Guided by the recent advancements in HTP and AI and an increasing need to accelerate the rate of genetic gain in peanut, the primary focus of this study revolves around harnessing the power of overt and latent phenotypes as well as ML methodologies for the accurate prediction of peanut yield. We hypothesize that ML models, incorporating an array of carefully extracted and selected traits in the form of latent phenotypes, will offer a robust prediction of yield in peanuts that plant breeders can implement into their programs. Therefore, the first objective of this study was i) to create high-resolution sigmoidal growth curves for important phenotypes, a mathematical representation of the peanut plant’s life cycle. Furthermore, we intended to derive growth-rate curves from these sigmoidal growth curves. Growth-rate curves, representing the first derivative of the sigmoidal curve and a visualization of the rate of change of growth over time, will offer valuable insights into the growth dynamics and vigor of the crop. As such, the second objective of this study was ii) to delve into the realm of hidden growth parameters by analyzing the generated growth curves and growth-rate curves to identify and extract latent phenotypes. The third and final objective of this study was iii) to select and use latent phenotypes in RF and XGBoost ML models to predict yield in peanuts and compare model performance.

## 2 Materials and methods

### 2.1 Germplasm and experimental design

**Figure 1:**
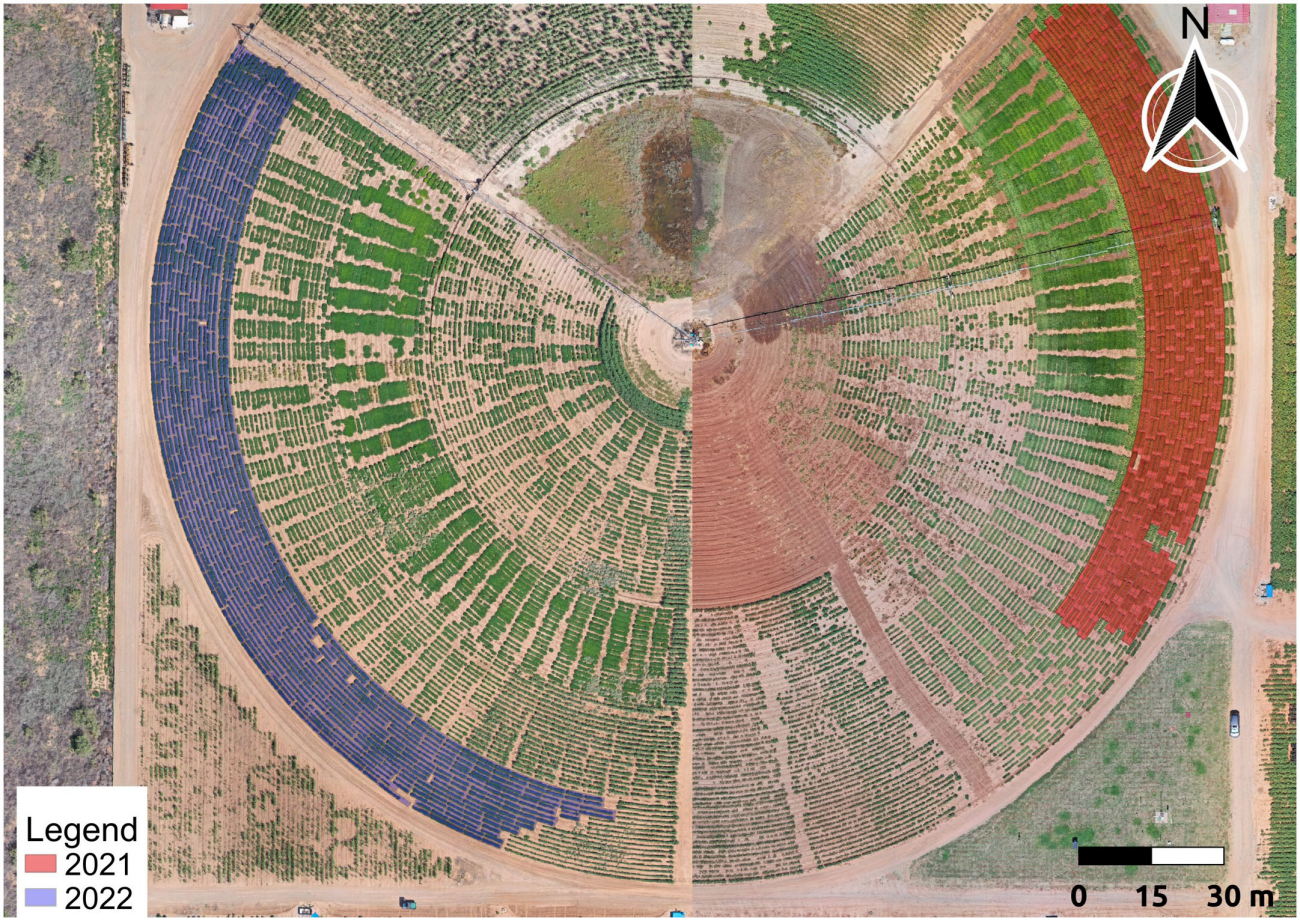
Maps of Experiment Area. These maps show an overview of the area of the experiments in Lubbock, TX in 2021 and 2022. The experiments were planted on the eastern and western sides of the same pivot-irrigated field. The red and purple regions indicate the 2021 and 2022 trials, respectively.

The germplasm used for this experiment consisted of variable material from four different experiments that shared a field location. There were 16 genotypes in an advanced breeding trial, 12 genotypes in a commercial variety trial, 12 genotypes from a population intended to study drought tolerance, and 40 top selections from a Recombinant Inbred Lines (RIL) population. However, because several genotypes were represented within multiple trials, the actual number of unique genotypes in the study was 73 rather than 80. Nonetheless, this population was quite diverse and was suitable for a yield prediction study due to how variable the material was. Each genotype within a test was represented across three replications in a randomized complete block design (RCBD), for a total of ~202 - 240 field plots each year. All phenotype extractions and yield predictions were conducted at the individual plot-level rather than across genotypes, with the exception of confusion matrices, which used the means for each genotype. The planted area for each plot measured approximately ~4.0m long by ~1.0m wide, and each plot consisted of two adjacent rows. The experiment was planted in a center pivot-irrigated field at the USDA-ARS Crop Stress Research Laboratory in Lubbock, TX (33°34′40′′ N, 101°53′24′′ W) in the summer of 2021 and 2022 (**Figure 1**). Standard agronomic practices for peanut were used while managing the plots as per the farm’s guidelines. Planting was done in May, and harvesting occurred approximately 130 days after sowing. Each entry in the trial was individually dug when it was considered to be at optimum maturity, determined using the hull-scrape method (referenced from Williams and Drexler, 1981). Peanut pods from each plot were dried using forced warm air to achieve a moisture level of around 10%. Subsequently, pod samples were cleaned before being weighed to determine yield.

### 2.2 Unmanned aerial vehicle flight missions and georeferencing

**Figure 2:**
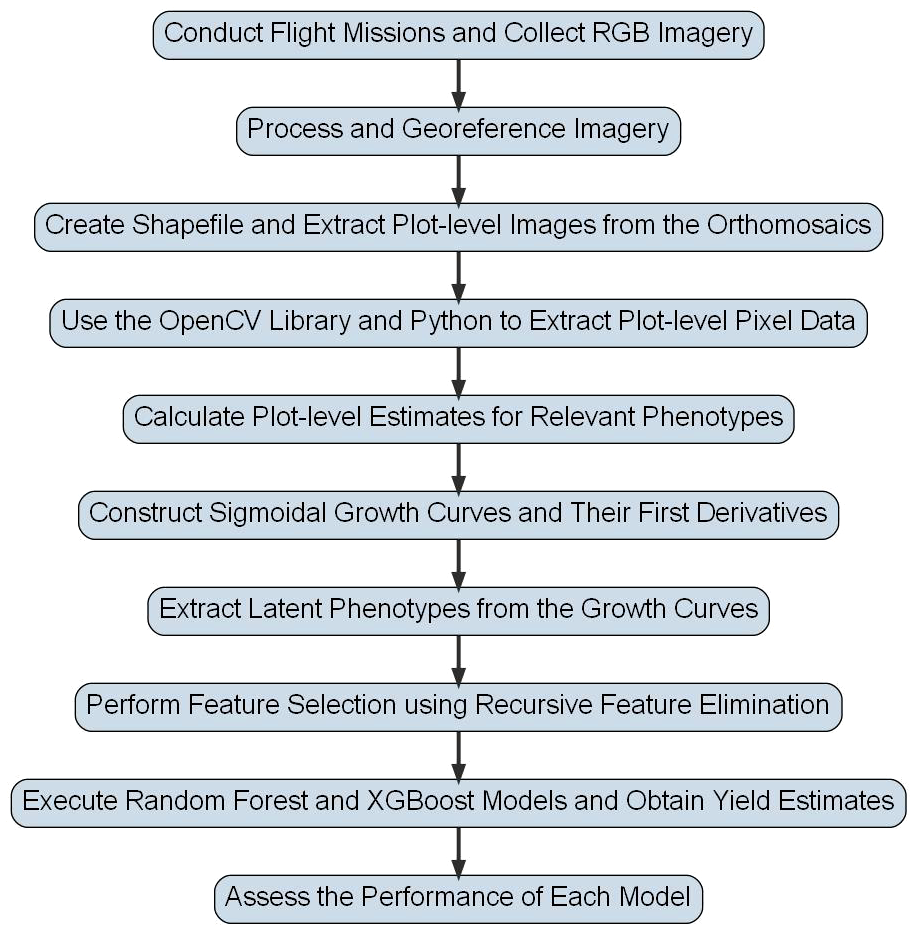
Flowchart for Peanut Yield Estimation Pipeline. This flowchart shows the general methodology used to derive yield estimates in this study. Steps have been simplified so that they can be visually presented, but detailed descriptions of each step are included in the text of this study.

A standard photogrammetry and machine learning workflow was used to produce the data used in this study (**Figure 2**). First, a series of unmanned aerial vehicle (UAV) flight missions were conducted throughout the summer of 2021 and 2022. The flight missions were conducted using a DJI Mavic 2 Pro equipped with the original payload, a (OEM) 20-megapixel RGB camera (DJI Industries, Shenzhen, China). At the 20-megapixel resolution, the ground sample distance (GSD) at 50m above ground level (AGL) was approximately 1.37 cm/pixel.

Flight missions were conducted at numerous time points throughout the season to generate high-resolution multitemporal growth curves. The 2021 season consisted of 12 UAV flights conducted over research plots that were made at an altitude of 50m AGL with 75% front image overlap and 65% - 70% side image overlap. The flight missions conducted in the 2022 season consisted of 18 UAV flights at a height of 50m with 75% front image overlap and 70% side image overlap. Unmanned aerial vehicle flights were conducted at or near solar noon (10AM-2PM CST). Flights were conducted on either cloudy or sunny days, and partly cloudy days were avoided since that could result in inconsistent cloud cover throughout a flight. Flights were corrected for brightness to account for differences between the cloudy and sunny days. After flight missions were conducted, raw image tiles were imported into Agisoft Metashape Professional for orthographic map image composition. Ground control points (GCPs) were collected using two paired Emlid Reach RS2+ (Emlid Tech Korlátolt, Budapest, Hungary) devices, where one device was placed on a fixed, permanent base location and the other was used as a rover to collect the GPS data for each GCP. Because prior research has demonstrated that four accurate and precise GCPs near the corners of each flight mission are sufficient to georeference photogrammetry projects, that is the number that was used in this study (Pugh et al., 2021).

### 2.3 Flight processing and feature extraction

Prior to processing and extraction, raw image tiles from each flight were examined to ensure that tiles erroneously collected before or after the flight mission(s) were removed. The imagery was processed in Agisoft Metashape Professional v. 2.0.1 (Agisoft, St. Petersburg, Russia). To estimate plant heights and derive 3-dimensional canopy volume, SfM was used to reconstruct the field. First, photograph alignment was conducted at the Highest quality setting, with a key point limit of 50,000 and a tie point limit of 25,000. The four GCPs were then added to each flight and cameras were optimized to ensure that projects were georeferenced. Point clouds were then constructed on the High-quality setting, which were used to produce the digital elevation maps (DEMs). Once High-quality DEMs were generated, high-resolution orthomosaics were constructed and exported for downstream feature extraction alongside the DEMs.

High-resolution orthomosaics and DEMs were imported into QGIS v. 3.22.4 software (QGIS Development Team, 2023), an open-source geographic information system (GIS) software package, for further plot-level analysis and extraction. The orthomosaics were aligned using a series of GCPs in the imagery that anchored them geographically, reducing the marginal error between scenes to ±5 cm. This precision allows for sequential time series-based image extractions using areas of measurement (AOMs) over plot-level image data (Young et al., 2020). These AOMs covered the plot area of the peanuts planted and had some slight border between the plots. The AOMs were rectangular, and their dimensions were approximately ~5m x 1m (~5m^2^) in 2021 and ~4m x 1m (~4m^2^) in 2022. After AOMs were placed over the orthomosaic layer, plot-level imagery was extracted from the final orthomosaic image for each plot and flight. This was accomplished using an iterative extraction process within QGIS. This was accomplished using the “Clip Raster by Mask Layer” tool. This brings up a selection menu for an “Input layer” encompassing the orthomosaic from each UAV flight and a “Mask layer” consisting of the plot-level AOMs. This iterative process then produced image extractions based on the plots that were placed over the orthomosaic images. This extraction workflow was used for each flight mission in 2021 and 2022.

### 2.4 Photogrammetric image processing

Plot-level imagery was extracted from 12 UAV flights in the 2021 season, resulting in approximately 6,000 plot-level images, while 18 UAV flights from the 2022 season resulted in approximately 14,000. These images consisted of RGB image data and ranged from ~100-400 kb in size each. The processing of this image data was done with Python (Python ver. 3.8.16) and the ‘OpenCV’ (OpenCV ver. 4.7.0) library (Bradski, 2000). Prior to any image analysis and extraction of the images, a method known as Contrast Limited Adaptive Histogram Equalization (CLAHE) was applied to the imagery (Reza, 2004). The flights were conducted under different lighting conditions, and the CLAHE method helped to correct the white balance in the images, particularly if the brightness varied across a single image. The imagery was then analyzed using the binary image masking threshold described by Young et al. (2020) and was used to separate the plants from the soil background. The binary image masking method was combined with the HSV (Hue, Saturation, Value) image threshold method to improve peanut plant masking. Hue, Saturation, and Value image thresholding takes the RGB image and converts it into the HSV color space, where Hue is the color, Saturation is the intensity of color, and Value is the brightness of the color. The next step in this process was to set an upper and lower HSV boundary representing the material in the imagery. The lower and upper boundaries used for the peanut plots in this study were (20, 0, 0) and (170, 255, 255), respectively; these values were determined on the range that the peanut plots reside in based on observation of the imagery and comparison of hue values with canopy pixels. This technique was combined with the binary image threshold to make an image mask that set any plant material in this range to 255 (white) and 0 (black) for the soil background. After the binary image mask was made, the relevant material in the image that was set to 255 was extracted from the original image and saved for further analysis. The resulting image from this operation was visible, segmented objects (peanut plants), and the rest was left blank (soil). The primary focus of this image extraction technique was to use the sequential plot imagery to develop plant canopy cover estimates (CC), canopy height estimates (CH), canopy volume estimates (CV), and to calculate an excess green index (ExG) (Chang et al., 2021). Canopy cover refers to the amount of each field plot taken up by the plant material, which has been shown to be valuable in prior studies (Lu et al., 2021). Plant height, or CH, has been shown to have excellent repeatability when estimated using UAV as compared to ground-based, conventional measurements using meter sticks (Pugh et al., 2018). Canopy volume is another parameter that has been shown to be valuable when estimated via UAV data, as it is simply the CH multiplied by the CC (Chang et al., 2021). Excess green index (ExG) is a vegetation index that has been demonstrated to work particularly well in the absence of multispectral data (Soontranon et al., 2014; Chang et al., 2021).

### 2.5 Calculation of crop phenotypes from processed photogrammetry products

The calculation of the plant-based features from the processed UAV imagery was accomplished using image analysis features provided in Python and the ‘OpenCV’ library. The Canopy Cover (CC) estimates were calculated using the methodology described in Chang et al. (2021). Canopy height (CH) estimates were produced using data that were extracted from the DEM map files processed from the UAV imagery data. This was accomplished by taking the modified images and applying the plant-level threshold to the DEM maps. Digital elevation map data were sequentially extracted using the plot-level extraction method described previously for each plot within each flight mission. After DEM data were extracted and the images were processed, the DEM data were segmented into deciles to be processed. Ultimately, the mean (CH) and 90^th^ percentile (CH90) of the DEM data were used for the CH estimates to determine which was most effective to use when including CH in a model.

Canopy volume (CV) estimates were calculated using the CC and CH estimates. The calculation was performed by simply multiplying the CC by the CH for each field plot. The CV was not indicative of a solid mass, due to the realities of canopy architecture, which mostly consists of open space. Nonetheless, CV estimates provided a usable metric for downstream analyses of crop growth. Because the mean and 90^th^ percentile data were extracted for CH, CH and CH90 were used to calculate CV, resulting in CV and CV90 metrics. The image data were RGB in this study due to a lack of multispectral sensors, thus a vegetation index was calculated with just the red and green spectral bands as an indicator of overall plant health. Excess Green Index (ExG) was calculated using the segmented image data for the image calculations as described in Chang et al. (2021). The ExG estimates were extracted from the images and then normalized to fit them to a simplified scale ranging from 0 to 1. The normalized mean ExG calculated from the image was extracted and used for subsequent analyses.

### 2.6 Data analysis, machine learning, and statistics

The photogrammetric data were systematically categorized based on the corresponding flight mission dates. Subsequent analyses were conducted to identify statistical anomalies, such as outliers; however, there was no obvious reason to remove the rare outlier cases and they were ultimately left in the dataset. Upon completion of this data structuring and cleaning process, Pearson’s correlation coefficients (*r*) were employed to determine the interrelationships between the four principal parameters (CC, CH, CV, and ExG) and the ultimate yield. These operations were executed using the ‘pandas’ library in Python (Van Rossum and Drake, 1995; McKinney, 2011).

**Figure 3:**
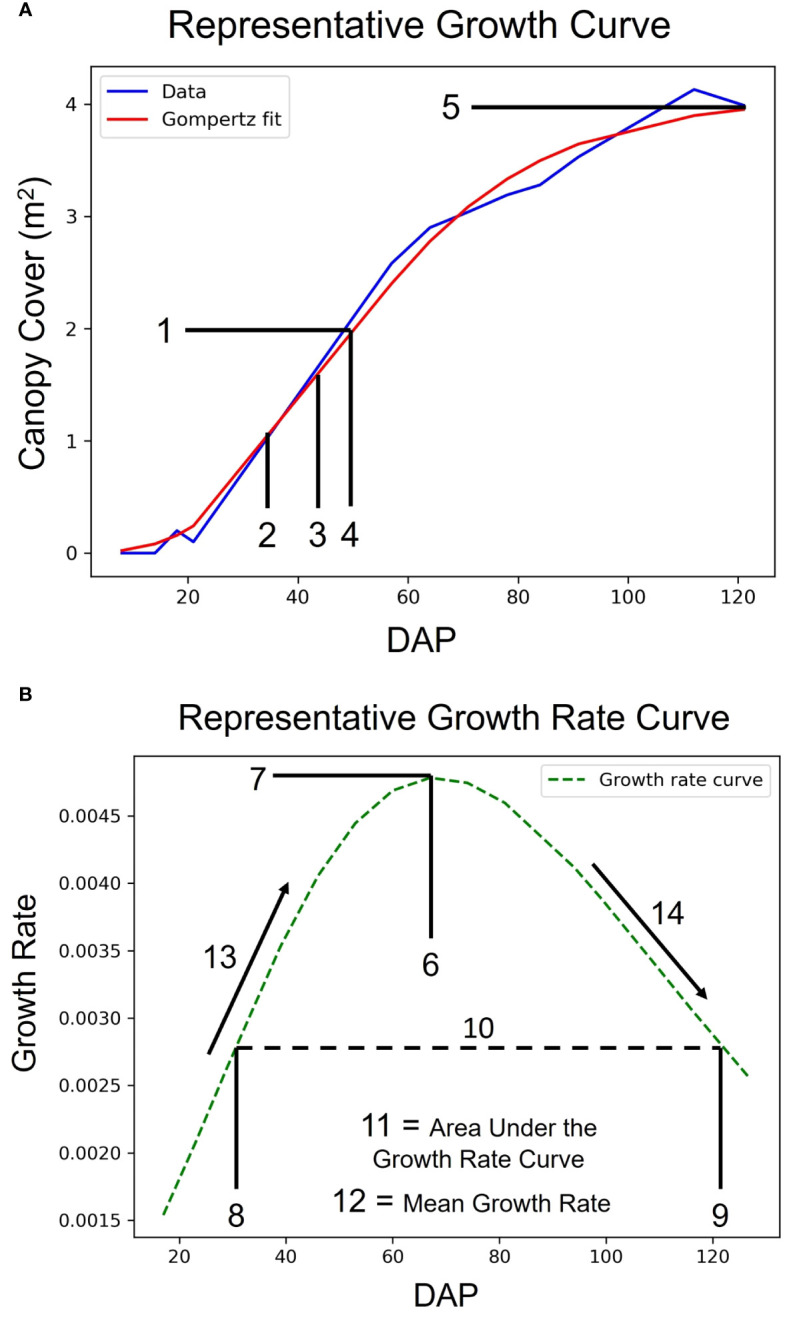
Growth Curves and Latent Phenotype Extraction. Demonstration of a representative sigmoidal growth curve **(A)** and a representative growth rate curve **(B)** as measures of traits vs. days after planting (DAP). The numbers denote latent phenotypes that were extracted from the curves. The array of features, in order, are: 1. Value of the Inflection Point, 2. DAP at 50% of Inflection Point, 3. DAP at 80% of Inflection Point, 4. DAP at Inflection Point, 5. Maximum Value, 6. DAP at Maximum Growth Rate, 7. Maximum Growth Rate, 8. DAP at First Half-maximum Growth Rate, 9. DAP at Last Half-maximum Growth Rate, 10. DAP Between Half-maximum Growth Rate, 11. Area under the Growth Rate Curve, 12. Mean Growth Rate, 13. Rate of Growth Rate Increase, and 14. Rate of Growth Rate Decrease.


Once Pearson’s correlations within each flight mission date were complete, high-resolution multitemporal growth curves as a function of each of the parameters vs DAP were generated for each field plot using the ‘SciPy’ and ‘matplotlib’ Python libraries (Barrett et al., 2005; Virtanen et al., 2020) (**Figure 3A**). Where necessary, due to rare situations where there were a few occluded plots on certain flight dates, imputations were made using the respective parameter means. This procedural step was imperative to ensure that each field plot possessed measurements corresponding to every flight mission date, thereby enabling the generation of congruent growth curves. Logistic and Gompertz sigmoidal functions were constructed so that they could be tested for their effectiveness in yield prediction; in addition, the first derivative of the sigmoidal curves, the growth rate curves, were also produced (**Figure 3B**) (Zwietering et al., 1990; Van Impe et al., 1992; Kucharavy and De Guio, 2015). Once these growth curves were generated and latent phenotypes could be extracted from them, the ML models could subsequently be executed with the extracted phenotypes. The equation for the logistic growth curve was:

$$N\left(t\right)=\;\frac{K}{1+\;\frac{K-N_{0}}{N_{0}}e^{-rt}}$$

where *N(t)* is the value of a metric at time *t*, *K* is the maximum value of the metric the plant(s) can sustain, *N_0_* is the initial value of the metric at *t = 0*, *r* is the intrinsic growth rate, and *e* is the base of natural logarithm. Gompertz sigmoidal growth curves were generated using the equation:


$$N\left(t\right)=N_{0}\text{exp}\left[-\beta e^{-\alpha t}\right]$$

where *N(t)* is the value of a metric at time *t*, *N_0_* is the initial value of the metric at *t* = 0, *α* is the growth rate parameter, *β* is related to the initial displacement of the growth curve, and *e* is the base of the natural logarithm. From the entire array of sigmoidal curves and their derivatives, a set of 14 latent phenotypes were extracted that were to be tested for their use in a yield prediction model. These latent phenotypes had either been used in previous, similar studies or were new features that we hypothesized could potentially be useful in a machine learning model (Chang et al., 2021).

Following latent phenotype extraction from the growth curves, an analysis using variance inflation factor (VIF) was conducted on the data using the ‘statsmodels’ library in Python (Seabold and Perktold, 2010; Akinwande et al., 2015). This is a commonly used metric which is calculated using the equation:


$$VIF=\;\frac{1}{1-\;R^{2}}$$

where *R^2^* is the coefficient of determination when a linear regression is performed between one variable upon all the other variables. Variance inflation factor can be included in regression analyses to determine if there is multicollinearity present in the data (Kim, 2019). Any latent phenotypes that had VIF scores greater than 5 were removed from the data before downstream analyses were conducted, to ensure that features with high multicollinearity would not fruitlessly be included in ML models (Kim, 2019).

After feature extraction using the growth models and feature exclusion via VIF, the remaining latent phenotypes were used to perform recursive feature elimination (RFE) using the ‘Scikit-learn’ library in Python (Pedregosa et al., 2011; Hao and Ho, 2019). This is a feature selection method which functions by recursively removing the least important features based upon a relative ranking of their importance. Through this process, RFE identifies and retains the most informative features for a given model. In this case, RFE was used to select features that were potentially useful for estimating final yield. To determine which configuration of latent phenotypes could best predict final yields, various limits of maximum selected features were specified from 1 – 30 and the various resulting feature configurations were tested in the ML scripts (Tunca et al., 2023). This iterative process ensured that an effective model could be built while minimizing complexity (Demir and Sahin, 2023).

Advanced machine learning techniques were employed to predict yield based on features identified through RFE. The Random Forest algorithm was the first ML method that was utilized via the ‘Scikit-learn’ library in Python (Pedregosa et al., 2011; Liu et al., 2012; Belgiu and Drăguţ, 2016). This algorithm generates an ensemble of decision trees during its training phase and subsequently predicts outcomes based on the average values derived from these individual trees (Dang et al., 2021; Guan et al., 2022). The unique characteristic of RF is its ability to train each decision tree on a distinct subset of the data, while also considering a randomized subset of features during each split. Such inherent randomness enhances the model’s resilience and reduces its susceptibility to overfitting (Liu et al., 2012). The collective outputs from all trees within the Random Forest are aggregated to yield the final prediction. The second ML algorithm that was tested in this study was eXtreme Gradient Boosting, or XGBoost, using the ‘Scikit-learn’ and ‘xgboost’ libraries in Python (Pedregosa et al., 2011; Chen and Guestrin, 2016). EXtreme Gradient Boosting is an advanced ensemble learning technique within a broader ecosystem of machine learning methodologies. Central to XGBoost is its iterative approach, wherein subsequent models are incorporated into the ensemble with the intent of rectifying inaccuracies present in prior models. This iterative refinement is facilitated by gradient boosting, wherein new models are tailored to the gradient of the loss function relative to the predictions of the current ensemble. Extreme Gradient Boosting is distinguished by its array of computational optimizations, encompassing regularization to mitigate overfitting, adept handling of absent data, and the capability for parallel tree construction, enhancing its computational efficiency (Herdter Smith, 2019). The workstation used to build these models was equipped with an Intel® Xeon® Gold 5218R CPU with 64.0 GB of installed RAM and an NVIDIA® RTX A5000 GPU.

Hyperparameter optimization was conducted for RF and XGBoost using the grid search method via ‘Scikit-learn’ (Pedregosa et al., 2011; Belete and Huchaiah, 2022). This systematic approach allows the user to examine model performance when using various combinations of hyperparameter settings via a specified “grid” of potential values, which allows users to efficiently identify and optimize hyperparameter settings for their data (Belete and Huchaiah, 2022). Once the grid search was performed, the optimal hyperparameters to use for each model were identified and used to execute the algorithms. For the RF model, the maximum depth of the decision trees was set to 10 and the number of estimators was set to 400. For the XGBoost model, the optimal learning rate was determined to be 0.06, the maximum depth was 3, and the minimum child weight was set to 5. Then, 1000 different random states, or iterations, were executed using Monte Carlo Cross-Validation (MCCV) for each model. Monte Carlo Cross-Validation was conducted to assess the models when using varying sizes for training and test sets and avoid the use of a non-robust model. As such, for each iteration, all the data were randomly separated into either the training set or the test set. To further assess the models, K-Fold Cross-Validation was performed with 10 splits so that multiple methods of cross-validation were tested. In addition, 70% and 30% of the data were randomly chosen for the training and test sets, respectively, and 1000 iterations were executed again for the 30 configurations obtained from the RFE processing described earlier. From these results, the best configuration and overall random state according to the training and test adjusted R^2^ scores was used for the predicted yield values present in the rest of this study.

Upon discerning the most refined RF and XGBoost models—characterized by their mean test and training adjusted R^2^ values—the model from the random state that had the highest training and test adjusted R^2^ values was used to algorithmically estimate yield for each plot. These yield predictions were subjected to regression analysis against the empirically measured yield from the field. This analytical step was important to gauge the precision and accuracy of the model’s predictions across the entirety of the dataset, beyond the confines of merely the test and training subsets, as demonstrated by Tunca et al. (2023). Consequently, the regression R^2^ was anticipated to have a value intermediary to the test and training metrics (Tunca et al., 2023). Regressions were also performed within each year of the study to determine model consistency, since years with vastly different mean yields and levels of variance could lead to inflation of coefficients of determination across years, i.e., two general “groupings” of the data resulting from highly variable environments could serve to anchor one another and artificially increase linear regression R^2^ values. To further evaluate the ability to increase the genetic gain rate for peanut yield using ML, repeatability (*R*) estimates were calculated within each year for each ML model. Because there was no familial structure across the material used for this study, it was not technically correct to calculate broad-sense heritability (*H^2^*) for the traits; however, repeatability is calculated in a similar fashion and can be used as a reasonable alternative metric for evaluation purposes. Repeatability estimates were calculated using the same all-random model and methodology as the one presented in Pugh et al. (2018). Next, to further evaluate the practicality and relevance of the predictions rendered by the machine learning algorithms, confusion matrices were constructed for each model, both intra-annually and inter-annually. This was accomplished using the ‘Scikit-learn’ and ‘matplotlib’ libraries within the Python framework (Barrett et al., 2005; Pedregosa et al., 2011).

Confusion matrices serve as a statistical tool designed to assess the efficacy of classification algorithms. These matrices facilitate the discernment of accurate classifications and serve to identify and estimate the degree of Type I and Type II errors, known colloquially as “false positives” and “false negatives”, respectively (Ruuska et al., 2018; Sharif et al., 2018). Notwithstanding its conventional application to classification endeavors, specifically pertaining to categorical variables, this method was employed to evaluate the prospective utility of Random Forest (RF) and eXtreme Gradient Boosting (XGBoost) within crop improvement programs. To investigate this, a quartet of categorical yield data, or “bins”, were devised, wherein “Excellent” encompassed the uppermost 10% of genotypes, “Good” spanned the 11^th^ to 25^th^ percentile, “Mediocre” covered the 26th to 50th percentile, and “Poor” encapsulated genotypes that did not achieve yields within the top half and would be likely to be excluded from being advanced within a breeding program. The mean yield for each genotype, aggregated across its respective plots, was computed, and subsequently allocated to one of the categories. These constructed yield categories were used to ascertain the extent to which plant breeders and other scientists might leverage RF and/or XGBoost for selection within their programs.

## 3 Results

### 3.1 Pearson’s correlation coefficients for yield vs. extracted phenotypes

**Figure 4:**
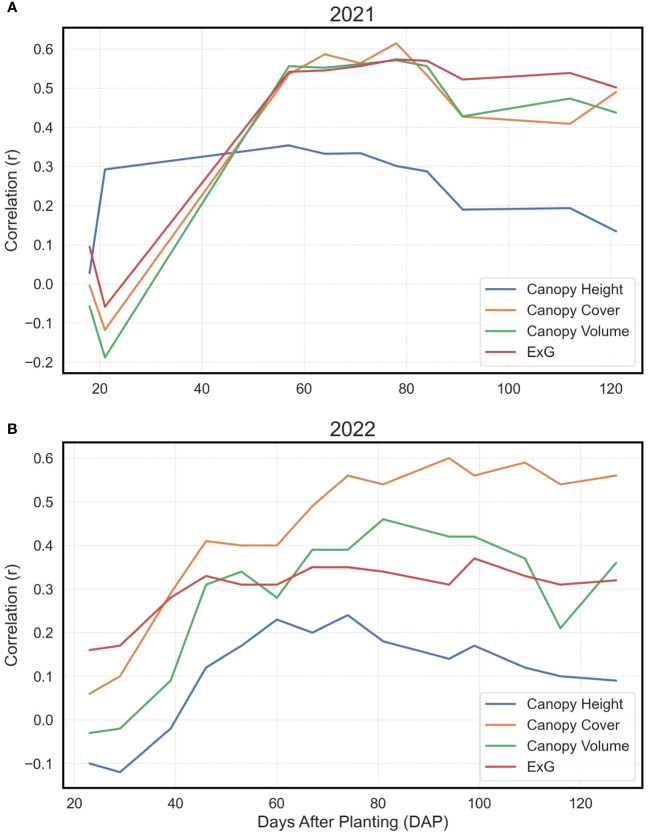
Pearson’s Correlations for Basic Extracted Traits vs. Yield. Depiction of Pearson’s correlation coefficients in 2021 **(A)** and 2022 **(B)** between Canopy Height, Canopy Cover, Canopy Volume, and Excess Green Index (ExG) with final yield at individual time points, shown as days after planting (DAP).

In the analysis, the Pearson’s correlation coefficients (*r*) delineating the relationship between directly procured phenotypes and yield exhibited analogous trends across both observational years (**Figure 4**). A notable increase in correlation magnitudes was observed within the temporal window of 20 to 60 days after planting (DAP). After this accelerated phase, the correlation coefficients largely stabilized, albeit with a minor decrement as the growing season approached its conclusion. Intriguingly, the canopy height parameter manifested a comparatively subdued correlation with yield relative to canopy cover, canopy volume, or ExG across both years. Among the evaluated traits, canopy cover consistently demonstrated the most robust correlation with yield over the years, mirroring the canopy volume and ExG performance in 2021, and surpassing both metrics in 2022.

### 3.2 Feature selection and correlations between selected features and yield

**Figure 5:**
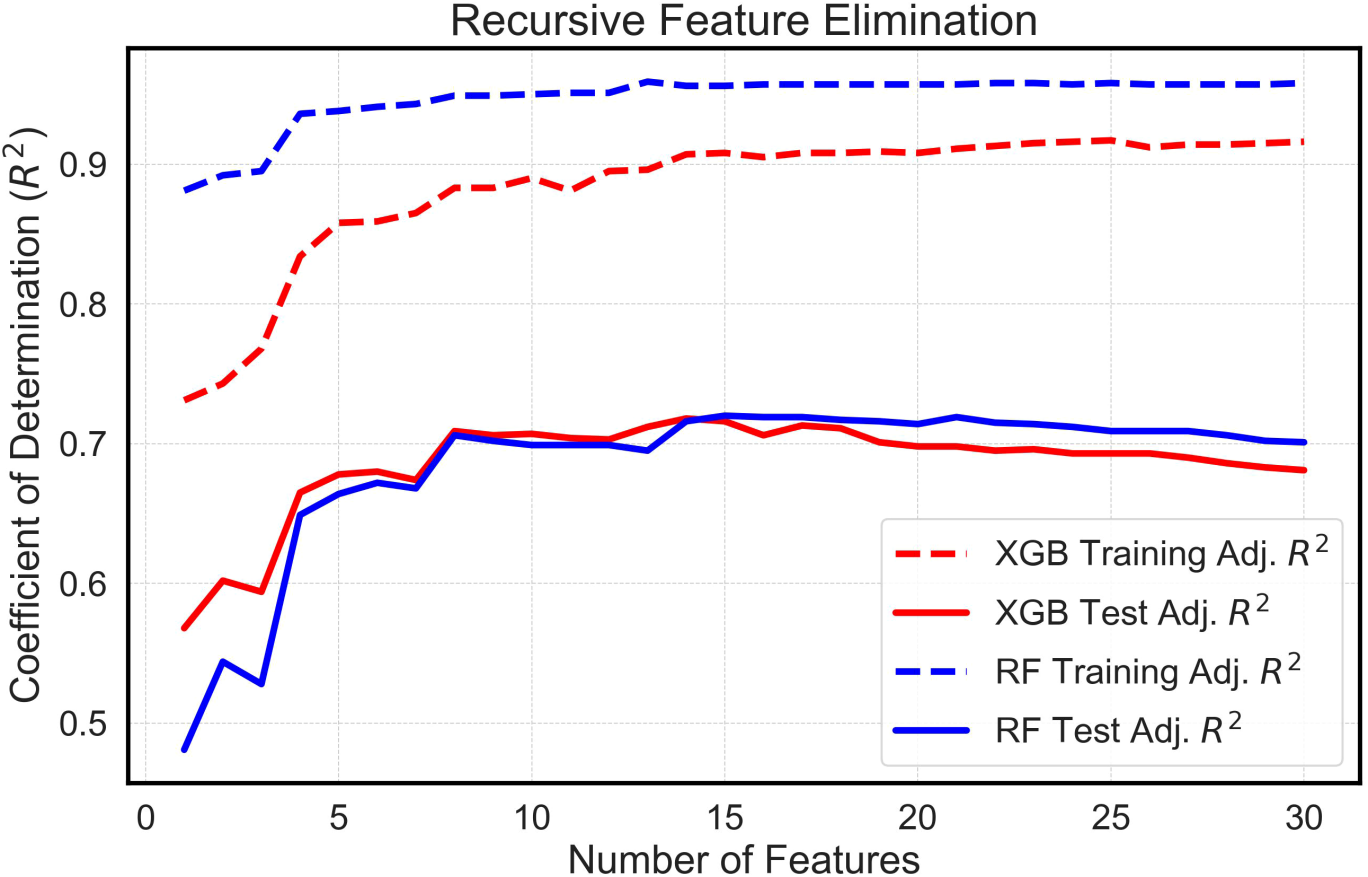
Feature Selection Using Recursive Feature Elimination. This is a visual depiction of feature selection using Recursive Feature Elimination (RFE). The graph shows the mean training and test adjusted Coefficient of Determination (R^2^) vs. the Number of Features. The R^2^ values derive from running numerous random states of the XGBoost (XGB) and Random Forest (RF) machine learning models.

The analysis systematically evaluated feature sets extracted from multitemporal growth trajectories and corresponding growth rate curves, encompassing a range of 1 to 30 features. Comparative assessments of these feature sets, when integrated with both the eXtreme Gradient Boosting (XGBoost) and Random Forest (RF) algorithms, yielded analogous outcomes (**Figure 5**). An examination of the models allowed for the identification of a threshold wherein the apex of mean adjusted training and test R^2^ values was attained with the least number of features. Interestingly, both algorithms required nearly identical optimized feature counts: 15 for the RF model and 14 for XGBoost. Furthermore, features extracted from the Gompertz growth model were discerned to exhibit superior predictive abilities compared to those extracted from the logistic model, because the RFE process always chose Gompertz-derived latent phenotypes over those derived from the logistic model. This consistently led to the exclusion of features derived from logistic growth curves in the finalized models. In addition, the RFE process determined that mean plant height was not valuable in the models and favored the use of the 90^th^ percentile of plant height estimates, instead. Consequently, these optimized feature configurations were employed in comparing yields estimated *in silico* by machine learning algorithms with the empirically measured yields recorded *in situ*.

**Figure 6:**
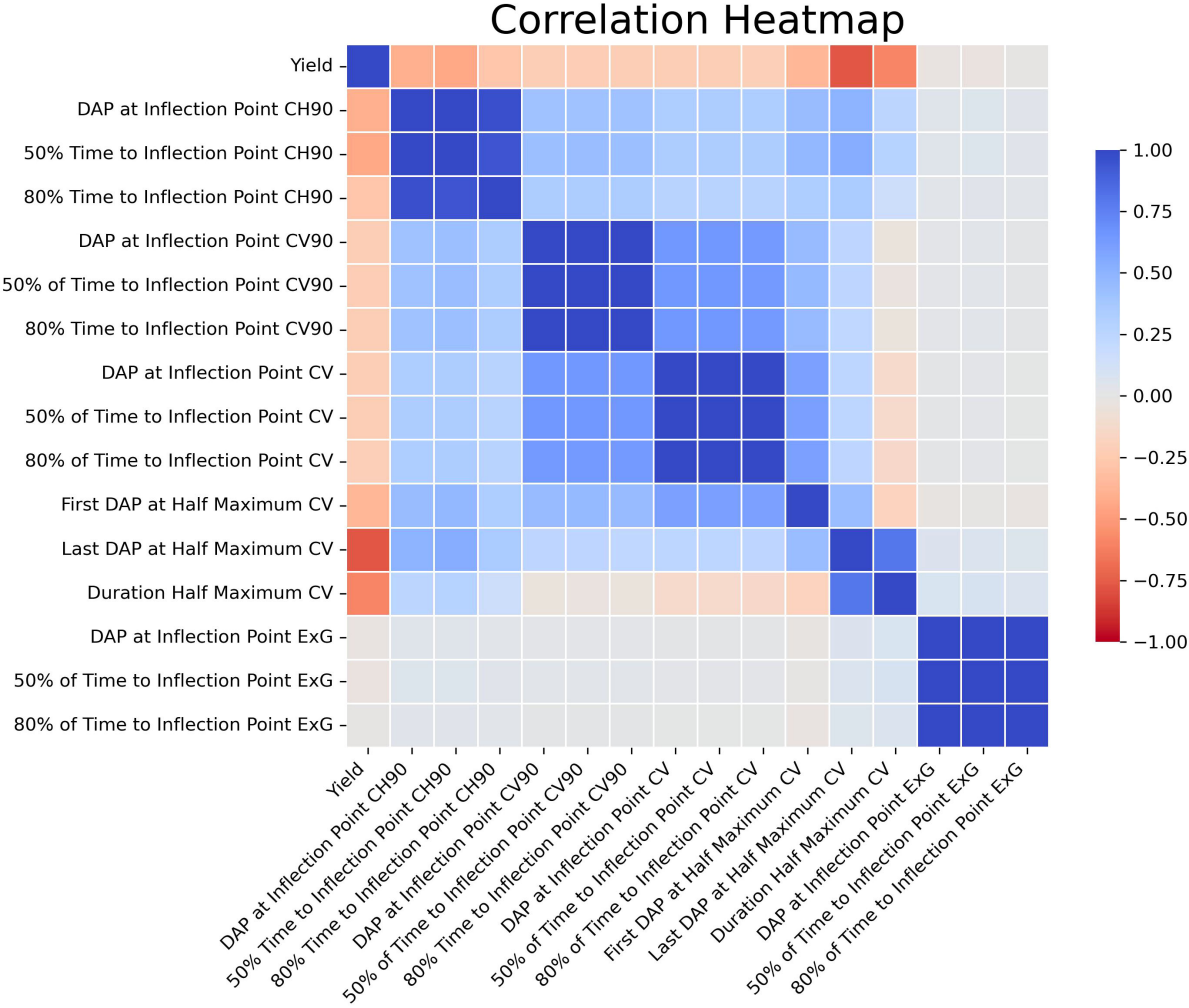
Correlation Heatmap for Latent Phenotypes. This is a visual representation of the Pearson’s correlation coefficients for the latent phenotypes selected via RFE vs yield. Values range from deep red (-1.00) to deep blue (1.00).

The 15 chosen features showed varying levels of independent correlation with the final yield (**Figure 6**). It is also important to note that there were strong correlations between several of the latent phenotypes with each other; indeed, in several cases Pearson’s correlations were approaching *r* = 1.00. Nonetheless, the features selected by RFE were used for this study due to potential relationships that may exist even between highly correlated traits that humans cannot feasibly anticipate or recognize. Since traits previously identified by VIF as collinear were already removed prior to these analyses, it was not determined that any remaining strongly correlated features needed to be removed from the ML models. The results of the MCCV analyses mirrored those seen when conducting the refinement via RFE. For the RF model, the MCCV adj. training and test R2 values were 0.96 and 0.72, respectively. For the XGBoost model, the MCCV adj. training and test R2 values were 0.91 and 0.72, respectively. Similarly, the K-fold CV adj. test R2 was 0.63 for both models. The Last DAP at Half Maximum Canopy Volume (CV) had the strongest relationship with yield on its own). Although there were strong positive correlations between many of the derived features with each other, the correlations between latent phenotypes and yield were overwhelmingly negative. No solitary latent phenotypes could be used to reliably predict yield on their own when comparing the entirety of the data across both years, and the data demonstrated that a model that incorporates multiple traits to arrive at predictions was necessary, corroborating the need for more complex ML models.

### 3.3 Regressions between estimated and measured yield and repeatability scores

**Figure 7:**
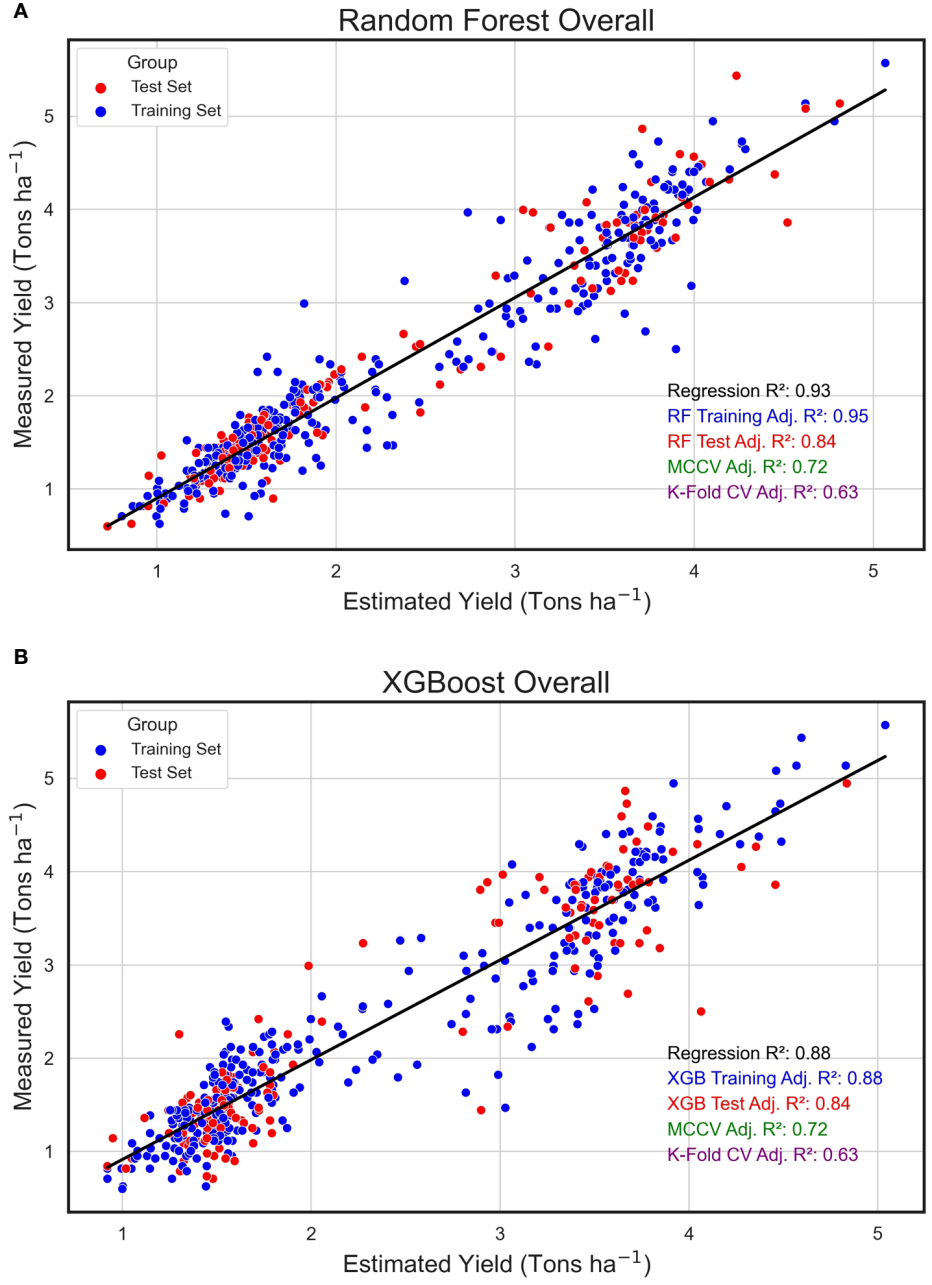
Overall Regression for Estimated and Measured Peanut Yield. These are linear regressions between plot-level yield values (Tons ha^-1^) predicted by machine learning models (Estimated Yield) and actual yield measured in the field (Measured Yield) when both years of the study were combined. The machine learning models used include random forest (RF, **A**) and XGBoost (XGB, **B**). Blue points represent field plots belonging to the training set and red dots represent plots that were used in the test set for each model. The adjusted training (blue) and test (red) adjusted R^2^ values for each model are included.

In an evaluation across all field plots and years, the Random Forest algorithm exhibited an impressive training adjusted R^2^ of 0.95, complemented by a test adjusted R^2^ of 0.84, signifying an optimal fit to the training dataset and alignment with the test data. Upon scrutinizing the regression between the algorithmically predicted yield values and the empirically measured yield in the field, the estimated data showed a robust relationship with the measured yield, evidenced by an R^2^ of 0.93 (**Figure 7A**). The eXtreme Gradient Boosting algorithm, while delivering a respectable performance in yield prediction, had a marginally reduced training R^2^ of 0.89 (**Figure 7B**). Furthermore, the relationship between its estimated and actual yield values, with an R^2^ of 0.88, was somewhat reduced compared to the yield estimates derived from the RF model.

**Figure 8:**
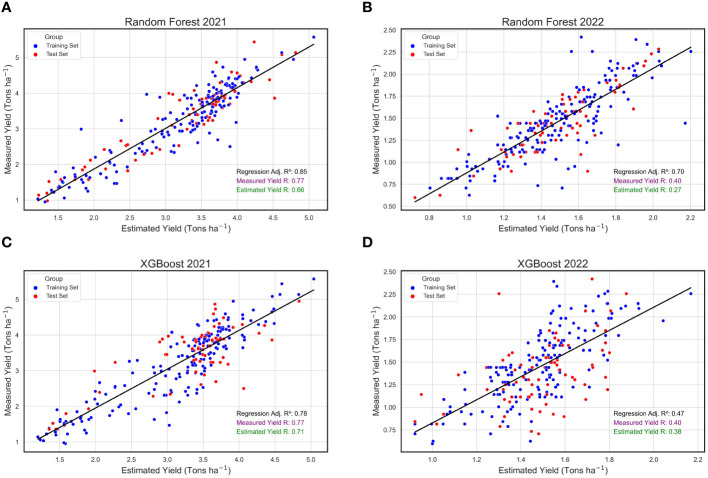
Annual Regressions for Estimated and Measured Peanut Yield. These are linear regressions between plot-level yield values (Tons ha^-1^) predicted by machine learning models (Estimated Yield) and actual yield measured in the field (Measured Yield) within the years 2021 2022. The machine learning models used include random forest (RF) in 2021 **(A)** and 2022 **(B)**, and XGBoost (XGB) in 2021 **(C)** and 2022 **(D)**. Blue points represent field plots belonging to the training set and red dots represent plots that were used in the test set for each model, and repeatability (*R*) scores are provided for the measured yield (purple) and estimated yield (green).

When analyzing the annual datasets independently, the RF algorithm exhibited relatively consistent performance across the two consecutive years under study, in contrast to the XGBoost algorithm. Specifically, the RF algorithm yielded an R^2^ of 0.85 in 2021, slightly decreasing to 0.70 in 2022 (**Figures 8A, B**). While these values were marginally reduced compared to the combined annual analysis, they remained reasonably high, underscoring the model’s robustness across diverse environmental conditions. Similarly, the XGBoost algorithm’s predicted yield values showed a strong performance in 2021 with an R^2^ of 0.78 (**Figure 8C**). However, its effectiveness substantially diminished in 2022, registering an R^2^ of only 0.47 (**Figure 8D**). Extreme Gradient Boosting was less consistent in this study when compared to RF. Repeatability (*R*) estimates were higher in 2021 when using both models than in 2022 (**Figure 8**). Notably, predicted yield values produced by XGBoost had higher *R* values and were closer to the *R* scores for actual yield when compared to the *R* estimates for predicted yields produced by the RF algorithm.

### 3.4 Confusion matrices for estimated and measured yield

**Figure 9:**
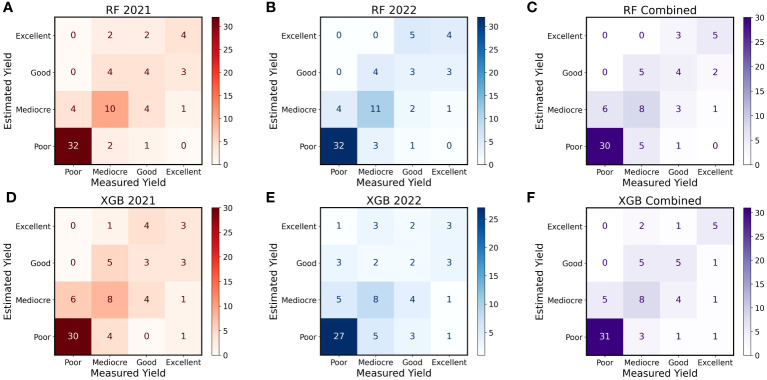
Confusion Matrices for Estimated and Measured Peanut Yield. These confusion matrices show the number of correct and incorrect categorizations of yield using Random Forest (RF) and XGBoost (XGB) models within and across 2021 and 2022. Shown, in order from left to right, are RF in 2021 **(A)**, RF in 2022 **(B)**, RF with the years combined **(C)**, XGB in 2021 **(D)**, XGB in 2022 **(E)**, and XGB with the years combined **(F)**. All genotypes were separated into four yield categories: Poor = bottom 50%, Mediocre = top 51 – 26%, Good = Top 25 – 11%, and Excellent = top 10% of genotypes. Genotypes were placed in each category using predictions from machine learning models (Estimated Yield) and estimates of yield collected in the field (Measured Yield). The number and shade of each square represents how many genotypes were placed in each category by each method. Genotypes placed in the exact same category by both methods will appear along the diagonal from lower left to upper right, and other squares represent varying degrees of incorrect classification.

In 2021 and 2022, the RF model performed better than the XGBoost model at separating the four different classes of yield data (**Figures 9A, B**). EXtreme gradient boosting performed much worse in 2022, with numerous strong misclassifications compared to RF (**Figure 9E**). In 2021, the two models performed more similarly; nonetheless, the Xgboost model still underperformed compared to RF due to larger number and severity of misclassifications (**Figures 9A, D**). This trend was slightly altered when examining classifications across both years as the amount and degree of Type I and II errors were reduced with both models. The RF model was able to identify Excellent and Good yielding genotypes more reliably than XGBoost, but both were reasonably effective at identifying Poor-yielding genotypes (**Figures 9C, F**).

## 4 Discussion

### 4.1 Overview and comparison to similar studies

For machine learning models to be useful in agricultural research, they must be able to reliably provide robust estimates of yield and other key parameters. Phenotypes extracted at an elementary level from field plots in this study and others have been directly correlated with yield, but none of these showed a strong or consistent enough correlation to be reliably predictive on their own, indicating the need for more sophisticated ML methodologies (Manley et al., 2023). Across the entire dataset, the performance of the RF and XGBoost models constructed in this study was superior to those seen in previous studies in peanut (Balota and Oakes, 2016; Bagherian et al., 2023; Shahi et al., 2023). In Balota and Oakes (2016), the R^2^ for yield was significantly lower than in the present study, ranging from 0.26 – 0.39. In Bagherian et al. (2023), the highest R^2^ achieved for estimating yield with Deep Learning (DL) and ML models was 0.61. Shahi et al. (2023) achieved similar R^2^ values to those reported in this study via RF; however, that study only considered one environment and had fewer field plots than the current study. Larger and more varied datasets have previously been demonstrated to lead to more accurate and robust ML predictions (Weitkamp and Karimi, 2023). Therefore, it is difficult to ascertain if the model(s) reported in Shahi et al. (2023) are as applicable to unseen data as the models presented here. In addition, it is important to note that several of these previous studies had the advantage of having access to spectral bands beyond the basic RGB. In contrast, this study only used an RGB camera while still achieving excellent results, which could be an important consideration for researchers and producers with limited access to multispectral sensors or who lack the requisite knowledge of how to use them (Sanches et al., 2018; Acorsi et al., 2019; Zeng et al., 2021). Indeed, the workflow presented here is achievable with widely accessible technology, as the latent phenotypes derived from Gompertz growth models produced using RGB data are the only variables used in the ML models (Borra-Serrano et al., 2020; Chang et al., 2021; Varela et al., 2021).

### 4.2 Random forest vs eXtreme gradient boosting

In this study, the Random Forest model was superior to eXtreme Gradient Boosting when examining the regressions between estimated and measured yield values, showing greater R^2^ values and more consistency across both years of the study. However, it is important to note that there was a large discrepancy in mean yields between 2021 and 2022; indeed, 2022 had much lower maximum yields and reduced variability between the top and bottom-yielding genotypes, which could partially explain the reduced effectiveness of the two models in that year. It is also critical to acknowledge that XGBoost had higher repeatability scores than RF in both years, which indicates that XGBoost may be better for actually capturing variation between genotypes and reducing the amount of unpartitioned error in the data. Because crop improvement efforts depend on the ability for the breeder to maximize genotypic variation and reduce error by accounting for different sources of error in the model, XGBoost may potentially be superior to RF when used as tool in a peanut breeding program in practice. In addition, XGBoost was developed and tailored to be computationally efficient to execute whereas RF does not place as much focus upon efficiency (Chen et al., 2015). In this study, the RF algorithm required a substantial amount of time to execute compared to XGBoost, often taking up to ~1 – 1.5 hours to execute its 1000 iterations on a high-end workstation intended for GIS and ML applications, whereas XGBoost could generally be completed in a matter of minutes. Nonetheless, the high degree of computational efficiency of XGBoost appears to have come at a significant cost to its raw predictive capabilities in this study, as demonstrated in the data. Therefore, it will be imperative that peanut breeders test various models and determine which ones will provide the best efficiency within their program; in the current study, there are legitimate cases to be made for either RF or XGBoost depending upon the situation.

It is also possible that XGBoost may improve at a faster rate than RF as additional, variable environments are added, since the performance of the two models was much more comparable when combining both years of data together. Once more environments and populations are added to the training data, it is likely that the higher repeatability of XGBoost will begin to emerge as a clear winner. However, this is speculation and would require additional environments to be added to test this hypothesis. With the presented data, RF is superior for producing consistently accurate yield estimates that produce high adjusted R^2^ values when regressed with yield collected in the field. These findings contrast with Tunca et al. (2023), where XGBoost was shown to be slightly more effective at predicting sorghum crop water content than RF. Perhaps more relevantly, these results also dispute the findings of Shahi et al. (2023), which showed that XGBoost was the ML model that produced the best predictions in peanut on its own. These discrepancies could be due to differences in the study population, the environments, the hyperparameters used, or a host of other potential factors. There are also studies where an RF algorithm effectively predicted traits of interest, corroborating these findings. In Joshi et al. (2023), RF performed better than XGBoost and Support Vector Machine (SVM) at predicting final wheat yields regardless of the input variable used. In Khan et al. (2022), maize yield prediction was most effective when using an RF model, outperforming the other methods tested. The variability in results across these studies indicates that the optimal model to use may be dependent on the population being studied, the environments the material is grown in, and the parameters being estimated (Bali and Singla, 2022). It is illogical to assume that there is a single “optimal” model across all conceivable situations. Testing a suite of different ML models may be necessary when attempting to build phenomic prediction algorithms to use within a peanut breeding program. Familiarity with the subject material, often referred to in the ML community as “domain knowledge,” will also be critical, and users will need to be cautious so that they do not become entirely reliant upon parameters estimated by ML algorithms without considering their context (Lischeid et al., 2022).

### 4.3 Machine learning for performing selection within a breeding program

One of the primary objectives of this study was to assess the value of RF and XGBoost for use within a peanut breeding program. While the regressions and repeatability scores demonstrate strong relationships between predicted and actual plot yields and a strong potential for improvement of yield using the ML outputs, the confusion matrices reveal more about the power of AI to enhance breeding programs. Perhaps one of the most important criteria for these models is to ensure they do not result in catastrophic selection errors, e.g., estimating Poor yield in an Excellent genotype, or vice versa. In this study, the only model that produced these selection errors was XGBoost, wherein one genotype was predicted to have Excellent yield (top 10% of genotypes) but was Poor (bottom 50% of genotypes) in actuality, and another genotype was identified as Excellent but was Poor. If we set the Excellent category as material that will be retained and the Poor category as material that will be excluded from a competitive peanut improvement program, this presents a clear problem. Effective rates of genetic gain require accurate phenotyping and prediction, and it is unlikely that repeated errors of this magnitude will be considered acceptable by most plant breeders (Dwivedi et al., 2020; Chen et al., 2022). Of course, such a prediction failure may be the result of damage to the crop in the field, accidental incomplete harvests, mistakes during data collection that lead to overestimated yields, and other errors. This could lead to predicted yields in an experiment failing to match final harvested yield through no fault of the ML models. Regardless, for simplicity, harvest yields should be the standard upon which we evaluate yield estimates generated *in silico*. In this case, XGBoost failed to match that standard in at least those two specific instances, despite its higher repeatability scores.

Fortunately, the ML models performed quite well outside of these two errors. While there were some occasional misidentifications, the models were reasonably able to correctly separate the genotypes into the four distinct yield categories. Remarkably, both models improved once the two years were combined. Once the years were considered together, the differences between RF and XGBoost greatly diminished, and both models could conceivably be implemented within a breeding program. Both models were able to reliably identify Poor genotypes that should be marked for removal from a program, one of the most important tasks for plant breeders. Inconsistencies in performance between 2021 and 2022 could be due to a host of factors, although the correlations of overt canopy characteristics and yield demonstrate that the correlations were overall much weaker in 2022 (**Figure 4**). This would naturally impair the function of ML models that predict yield based upon those canopy characteristics. It is well understood that ML models are much more robust and reliable when they have multiple, variable sets of data, e.g., environments, to train them (Ren et al., 2023). Thus, it is reasonable to hypothesize that both models would be further improved if additional locations were added to the data. In addition, the latent phenotypes presented in this study do not remotely encompass the multitude of variables that affect crop yields. One of the most important predictive components for crop yields that is absent in this study is direct quantitative information about the environment (Sudduth et al., 1996; Liliane and Charles, 2020). While the predicted yield values obtained in this study indirectly incorporate environment effects as a result of replication of genotypes within the field, no quantitative environmental or spatial data at the plot-level could be incorporated into the models as there were none of these data collected. Spatial soil and environmental data may be key elements that could greatly increase the efficacy of ML models (Pandith et al., 2020). Data for the presence of pests, the weather, the soil, and many other biotic and abiotic factors could assist ML models in producing reliable predictions (Sudduth et al., 1996; Pugh et al., 2019). Despite the absence of these data in the present study, our results demonstrate that peanut breeders and researchers can use RF and XGBoost models to make program selections if they use appropriate training data.

## 5 Conclusions

The fusion of remote sensing techniques with sophisticated machine learning (ML) algorithms promises transformative advancements for plant breeding programs. Once ML-driven workflows achieve sufficient accuracy and precision in yield predictions, the traditional practice of harvesting experimental plots to obtain yield measurements may become obsolete. Bypassing this labor-intensive step not only reduces financial inputs but also diminishes the reliance on resources, such as fossil fuels, required to obtain conventional yield measurements. The construction of resilient ML frameworks tailored for crops like peanuts mandates their calibration using data spanning diverse germplasm and an array of environmental contexts. The richness of training data directly amplifies ML algorithms’ universality and predictive prowess, enhancing their adaptability to novel and unforeseen scenarios.

Looking toward the horizon, it is imperative for scientists to explore a broader spectrum of ML architectures and to develop models that estimate vital parameters beyond yield. Such an endeavor requires a multidisciplinary confluence of experts from remote sensing, data analytics, plant breeding, among many other fields. Given the dynamic nature of machine learning as a discipline, a relentless pursuit of model evaluation and comparison is critical. Yet, the potential dividends from using these techniques in a crop improvement program eclipses the associated investment in model development and validation. In summary, this investigation demonstrates the profound potential of predictive machine learning frameworks in peanut breeding programs.

## Data availability statement

The raw data supporting the conclusions of this article will be made available by the authors, without undue reservation.

## Author contributions

NAP: Conceptualization, Data curation, Formal analysis, Investigation, Methodology, Project administration, Software, Supervision, Validation, Visualization, Writing – original draft, Writing – review & editing. AY: Conceptualization, Data curation, Formal analysis, Investigation, Methodology, Software, Visualization, Writing – original draft, Writing – review & editing. MO: Data curation, Investigation, Writing – review & editing. YE: Data curation, Resources, Writing – review & editing. JS: Data curation, Investigation, Resources, Writing – review & editing. ZX: Resources, Supervision, Writing – review & editing. NP: Conceptualization, Data curation, Funding acquisition, Project administration, Resources, Writing – review & editing.

## References

[B1] Acorsi M. G. das Dores Abati Miranda F. Martello M. Smaniotto D. A. Sartor L. R. (2019). Estimating biomass of black oat using UAV-based RGB imaging. Agronomy 9, 344. doi: 10.3390/agronomy9070344

[B2] Akinwande M. O. Dikko H. G. Samson A. (2015). Variance inflation factor: as a condition for the inclusion of suppressor variable (s) in regression analysis. Open J. Stat 5, 754. doi: 10.4236/ojs.2015.57075

[B3] Araus J. L. Kefauver S. C. Zaman-Allah M. Olsen M. S. Cairns J. E. (2018). Translating high-throughput phenotyping into genetic gain. Trends Plant Sci. 23, 451–466. doi: 10.1016/j.tplants.2018.02.001 29555431 PMC5931794

[B4] Ashapure A. Jung J. Chang A. Oh S. Yeom J. Maeda M.. (2020). Developing a machine learning based cotton yield estimation framework using multi-temporal UAS data. ISPRS J. Photogram. Remote Sens. 169, 180–194. doi: 10.1016/j.isprsjprs.2020.09.015

[B5] Awal M. A. Ikeda T. (2003). Controlling canopy formation, flowering, and yield in field-grown stands of peanut (Arachis hypogaea L.) with ambient and regulated soil temperature. Field Crops Res. 81, 121–132. doi: 10.1016/S0378-4290(02)00216-2

[B6] Bagherian K. Bidese-Puhl R. Bao Y. Zhang Q. Sanz-Saez A. Dang P. M.. (2023). Phenotyping agronomic and physiological traits in peanut under mid-season drought stress using UAV-based hyperspectral imaging and machine learning. Plant Phenome J. 6, e20081. doi: 10.1002/ppj2.20081

[B7] Bali N. Singla A. (2022). Emerging trends in machine learning to predict crop yield and study its influential factors: A survey. Arch. Comput. Methods Eng. 1-18. doi: 10.1007/s11831-021-09569-8

[B8] Balota M. Oakes J. (2016). “Exploratory use of a UAV platform for variety selection in peanut,” in Autonomous Air and Ground Sensing Systems for Agricultural Optimization and Phenotyping, vol. 9866. (SPIE), 54–62.

[B9] Barrett P. Hunter J. Miller J. T. Hsu J. C. Greenfield P. (2005). “matplotlib–A Portable Python Plotting Package,” in Astronomical data analysis software and systems XIV, vol. 347, 91.

[B10] Belete D. M. Huchaiah M. D. (2022). Grid search in hyperparameter optimization of machine learning models for prediction of HIV/AIDS test results. Int. J. Comput. Appl. 44 (9), 875–886.

[B11] Belgiu M. Drăguţ L. (2016). Random forest in remote sensing: A review of applications and future directions. ISPRS J. photogram. Remote Sens. 114, 24–31. doi: 10.1016/j.isprsjprs.2016.01.011

[B12] Boote K. J. (1982). Growth stages of peanut (Arachis hypogaea L.). Peanut Sci. 9, 35–40. doi: 10.3146/i0095-3679-9-1-11

[B13] Borra-Serrano I. De Swaef T. Quataert P. Aper J. Saleem A. Saeys W.. (2020). Closing the phenotyping gap: High resolution UAV time series for soybean growth analysis provides objective data from field trials. Remote Sens. 12, 1644. doi: 10.3390/rs12101644

[B14] Bradski G. (2000). The openCV library. Dr. Dobb’s Journal: Softw. Tools Prof. Program. 25, 120–123.

[B15] Breiman L. (2001). Random forests. Mach. Learn. 45, 5–32. doi: 10.1023/A:1010933404324

[B16] Bustos-Korts D. Boer M. P. Malosetti M. Chapman S. Chenu K. Zheng B.. (2019). Combining crop growth modeling and statistical genetic modeling to evaluate phenotyping strategies. Front. Plant Sci. 10, 1491. doi: 10.3389/fpls.2019.01491 31827479 PMC6890853

[B17] Carneiro F. M. Furlani C. E. Zerbato C. Menezes P. C. D. Gírio L. A. D. S. (2019). Correlations among vegetation indices and peanut traits during different crop development stages. Engenharia Agrícola 39, 33–40. doi: 10.1590/1809-4430-eng.agric.v39nep33-40/2019

[B18] Chamberlin K. D. (2019). Not your grandma’s goobers: designing the future of peanut breeding. Peanut Sci. 46, 91–98. doi: 10.3146/0095-3679-46.1A.91

[B19] Chang A. Jung J. Yeom J. Maeda M. M. Landivar J. A. Enciso J. M.. (2021). Unmanned aircraft system-(UAS-) based high-throughput phenotyping (HTP) for tomato yield estimation. J. Sensors 2021, 1–14. doi: 10.1155/2021/5723567

[B20] Chen T. Guestrin C. (2016). “Xgboost: A scalable tree boosting system,” in Proceedings of the 22nd acm sigkdd international conference on knowledge discovery and data mining. 785–794.

[B21] Chen T. He T. Benesty M. Khotilovich V. Tang Y. Cho H.. (2015). Xgboost: extreme gradient boosting. R Package version 0.4-2 1, 1–4.

[B22] Chen C. J. Rutkoski J. Schnable J. C. Murray S. C. Wang L. Jin X.. (2022). Role of the genomics–phenomics–agronomy paradigm in plant breeding. Plant Breed. Rev. 46, 627–673. doi: 10.1002/9781119874157.ch10

[B23] Dang C. Liu Y. Yue H. Qian J. Zhu R. (2021). Autumn crop yield prediction using data-driven approaches: -support vector machines, random forest, and deep neural network methods. Can. J. Remote Sens. 47, 162–181. doi: 10.1080/07038992.2020.1833186

[B24] Davis J. P. Dean L. L. (2016). Peanut composition, flavor and nutrition. Peanuts: Gene. Process. Util., 289–345. doi: 10.1016/B978-1-63067-038-2.00011-3

[B25] Demir S. Sahin E. K. (2023). An investigation of feature selection methods for soil liquefaction prediction based on tree-based ensemble algorithms using AdaBoost, gradient boosting, and XGBoost. Neural Comput. Appl. 35, 3173–3190. doi: 10.1007/s00521-022-07856-4

[B26] Dwivedi S. L. Goldman I. Ceccarelli S. Ortiz R. (2020). Advanced analytics, phenomics and biotechnology approaches to enhance genetic gains in plant breeding. Adv. Agron. 162, 89–142. doi: 10.1016/bs.agron.2020.02.002

[B27] Faye B. Webber H. Diop M. Mbaye M. L. Owusu-Sekyere J. D. Naab J. B.. (2018). Potential impact of climate change on peanut yield in Senegal, West Africa. Field Crops Res. 219, 148–159. doi: 10.1016/J.FCR.2018.01.034

[B28] Feldmann M. J. Gage J. L. Turner-Hissong S. D. Ubbens J. R. (2021). Images carried before the fire: The power, promise, and responsibility of latent phenotyping in plants. Plant Phenome J. 4, e20023. doi: 10.1002/ppj2.20023

[B29] Fletcher S. M. Shi Z. (2016). An overview of world peanut markets. Peanuts, 267–287. doi: 10.1016/B978-1-63067-038-2.00010-1

[B30] Furbank R. T. Tester M. (2011). Phenomics–technologies to relieve the phenotyping bottleneck. Trends Plant Sci. 16, 635–644. doi: 10.1016/j.tplants.2011.09.005 22074787

[B31] Gage J. L. Richards E. Lepak N. Kaczmar N. Soman C. Chowdhary G.. (2019). In-field whole-plant maize architecture characterized by subcanopy rovers and latent space phenotyping. Plant Phenome J. 2, 1–11. doi: 10.2135/tppj2019.07.0011

[B32] Guan Y. Grote K. Schott J. Leverett K. (2022). Prediction of soil water content and electrical conductivity using random forest methods with UAV multispectral and ground-coupled geophysical data. Remote Sens. 14, 1023. doi: 10.3390/rs14041023

[B33] Haerani H. Apan A. Nguyen-Huy T. Basnet B. (2023). Modelling future spatial distribution of peanut crops in Australia under climate change scenarios. Geo-spatialInform. Sci., 1–20. doi: 10.1080/10095020.2022.2155255

[B34] Hao J. Ho T. K. (2019). Machine learning made easy: a review of scikit-learn package in python programming language. J. Educ. Behav. Stat 44, 348–361. doi: 10.3102/1076998619832248

[B35] Herdter Smith E. (2019). Using extreme gradient boosting (XGBoost) to evaluate the importance of a suite of environmental variables and to predict recruitment of young-of-the-year spotted seatrout in Florida. BioRxiv, 543181. doi: 10.1101/543181

[B36] Huang J. Gómez-Dans J. L. Huang H. Ma H. Wu Q. Lewis P. E.. (2019). Assimilation of remote sensing into crop growth models: Current status and perspectives. Agric. For. meteorol. 276, 107609. doi: 10.1016/j.agrformet.2019.06.008

[B37] Janni M. Pieruschka R. (2022). Plant phenotyping for a sustainable future. J. Exp. Bot. 73, 5085–5088. doi: 10.1093/jxb/erac286 36056763

[B38] Joshi A. Pradhan B. Chakraborty S. Behera M. D. (2023). Winter wheat yield prediction in the conterminous United States using solar induced chlorophyll fluorescence data and XGBoost and random forest algorithm. Ecol. Inf. 77, 102194. doi: 10.1016/j.ecoinf.2023.102194

[B39] Khan S. N. Li D. Maimaitijiang M. (2022). A geographically weighted random forest approach to predict corn yield in the US corn belt. Remote Sens. 14 (12), 2843.

[B40] Kim J. H. (2019). Multicollinearity and misleading statistical results. Korean J. anesthesiol. 72, 558–569. doi: 10.4097/kja.19087 31304696 PMC6900425

[B41] Kris-Etherton P. M. Pearson T. A. Wan Y. Hargrove R. L. Moriarty K. Fishell V.. (1999). High–monounsaturated fatty acid diets lower both plasma cholesterol and triacylglycerol concentrations. Am. J. Clin. Nutr. 70, 1009–1015. doi: 10.1093/ajcn/70.6.1009 10584045

[B42] Kucharavy D. De Guio R. (2015). Application of logistic growth curve. Proc. Eng. 131, 280–290. doi: 10.1016/j.proeng.2015.12.390

[B43] Lane H. M. Murray S. C. (2021). High throughput can produce better decisions than high accuracy when phenotyping plant populations. Crop Sci. 61, 3301–3313. doi: 10.1002/csc2.20514

[B44] Laza H. E. Acosta-Martinez V. Cano A. Baker J. Mahan J. Gitz D.. (2023). Elevated [CO2] enhances soil respiration and AMF abundance in a semiarid peanut agroecosystem. Agriculture Ecosyst. Environ. 355, 108592.

[B45] Laza H. E. Baker J. T. Yates C. Mahan J. R. Burow M. D. Puppala N.. (2021). Effect of elevated CO2 on peanut performance in a semi-arid production region. Agric. For. Meteorology 308, 108599.

[B46] Liliane T. N. Charles M. S. (2020). Factors affecting yield of crops. Agronomy-climate Change Food Secur. 9. doi: 10.5772/intechopen.90672

[B47] Lischeid G. Webber H. Sommer M. Nendel C. Ewert F. (2022). Machine learning in crop yield modelling: A powerful tool, but no surrogate for science. Agric. For. Meteorol. 312, 108698. doi: 10.1016/j.agrformet.2021.108698

[B48] Liu Y. Wang Y. Zhang J. (2012). “New machine learning algorithm: Random forest,” in Information Computing and Applications: Third International Conference, ICICA 2012, Chengde, China, September 14-16, 2012. 246–252 (Springer Berlin Heidelberg), Proceedings 3.

[B49] Lobos G. A. Camargo A. V. Del Pozo A. Araus J. L. Ortiz R. Doonan J. H. (2017). Plant phenotyping and phenomics for plant breeding. Front. Plant Sci. 8, 2181. doi: 10.3389/fpls.2017.02181 29375593 PMC5770690

[B50] Lu J. Cheng D. Geng C. Zhang Z. Xiang Y. Hu T. (2021). Combining plant height, canopy coverage and vegetation index from UAV-based RGB images to estimate leaf nitrogen concentration of summer maize. Biosyst. Eng. 202, 42–54. doi: 10.1016/j.biosystemseng.2020.11.010

[B51] Manley A. Ravelombola W. Cason J. Bennett B. Pham H. Kimura E.. (2023). Use of unmanned aerial system (UAS) phenotyping to predict pod and seed yield in organic peanuts. Am. J. Plant Sci. 14, 415–426. doi: 10.4236/ajps.2023.143027

[B52] Marshall M. Thenkabail P. (2015). Developing in *situ* non-destructive estimates of crop biomass to address issues of scale in remote sensing. Remote Sens. 7, 808–835. doi: 10.3390/rs70100808

[B53] McKinney W. (2011). pandas: a foundational Python library for data analysis and statistics. Python High Perform. Sci. comput. 14, 1–9.

[B54] Moctezuma E. Feldman L. J. (1999). Auxin redistributes upwards in graviresponding gynophores of the peanut plant. Planta 209, 180–186. doi: 10.1007/S004250050620/METRICS 10436219

[B55] Nazari L. Ghotbi V. Nadimi M. Paliwal J. (2023). A Novel Machine-Learning Approach to Predict Stress-Responsive Genes in Arabidopsis. 16 (9), 407.

[B56] Nijland W. De Jong R. De Jong S. M. Wulder M. A. Bater C. W. Coops N. C. (2014). Monitoring plant condition and phenology using infrared sensitive consumer grade digital cameras. Agric. For. Meteorol. 184, 98–106. doi: 10.1016/j.agrformet.2013.09.007

[B57] Obasa K. Haynes L. (2022). Two new bacterial pathogens of peanut, causing early seedling decline disease, identified in the Texas Panhandle. Plant Dis. 106, 648–653. doi: 10.1094/PDIS-07-21-1555-RE 34597146

[B58] Pandith V. Kour H. Singh S. Manhas J. Sharma V. (2020). Performance evaluation of machine learning techniques for mustard crop yield prediction from soil analysis. J. Sci. Res. 64, 394–398. doi: 10.37398/JSR.2020.640254

[B59] Pauli D. Andrade-Sanchez P. Carmo-Silva A. E. Gazave E. French A. N. Heun J.. (2016). Field-based high-throughput plant phenotyping reveals the temporal patterns of quantitative trait loci associated with stress-responsive traits in cotton. G3: Genes Genomes Genet. 6, 865–879. doi: 10.1534/g3.115.023515 PMC4825657 26818078

[B60] Pedregosa F. Varoquaux G. Gramfort A. Michel V. Thirion B. Grisel O.. (2011). Scikit-learn: machine learning in python. J. Mach. Learn. Res. 12, 2825–2830.

[B61] Pugh N. A. Horne D. W. Murray S. C. Carvalho J. G. Malambo L. Jung J.. (2018). Temporal estimates of crop growth in sorghum and maize breeding enabled by unmanned aerial systems. Plant Phenome J. 1, 1–10. doi: 10.2135/tppj2017.08.0006

[B62] Pugh N. A. Morgan C. L. Schnell R. Horn K. Pietsch D. Rooney W. L. (2019). A statistical evaluation of replicated block designs and spatial variability in sorghum performance trials. J. Crop Improve. 33, 551–566. doi: 10.1080/15427528.2019.1627686

[B63] Pugh N. A. Thorp K. R. Gonzalez E. M. Elshikha D. E. M. Pauli D. (2021). Comparison of image georeferencing strategies for agricultural applications of small unoccupied aircraft systems. Plant Phenome J. 4, e20026. doi: 10.1002/ppj2.20026

[B64] Puppala N. Nayak S. N. Sanz-Saez A. Chen C. Devi M. J. Nivedita N.. (2023). Sustaining yield and nutritional quality of peanuts in harsh environments: Physiological and molecular basis of drought and heat stress tolerance. Front. Genet. 14. doi: 10.3389/fgene.2023.1121462 PMC10030941 36968584

[B65] QGIS Development Team. (2023). QGIS Geographic Information System (Open Source Geospatial Foundation Project). Available at: http://qgis.osgeo.org.

[B66] Qi H. Zhu B. Wu Z. Liang Y. Li J. Wang L.. (2020). Estimation of peanut leaf area index from unmanned aerial vehicle multispectral images. Sensors 20, 6732. doi: 10.3390/s20236732 33255612 PMC7728055

[B67] Ren P. Li H. Han S. Chen R. Yang G. Yang H.. (2023). Estimation of soybean yield by combining maturity group information and unmanned aerial vehicle multi-sensor data using machine learning. Remote Sensing. 15, 4286. doi: 10.3390/rs15174286

[B68] Reza A. M. (2004). Realization of the contrast limited adaptive histogram equalization (CLAHE) for real-time image enhancement. J. VLSI Signal Process. Syst. signal image video Technol. 38, 35–44. doi: 10.1023/B:VLSI.0000028532.53893.82

[B69] Rouse J. W. Haas R. H. Schell J. A. Deering D. W. (1974). Monitoring vegetation systems in the Great Plains with ERTS. NASA Spec. Publ 351, 309.

[B70] Ruuska S. Hämäläinen W. Kajava S. Mughal M. Matilainen P. Mononen J. (2018). Evaluation of the confusion matrix method in the validation of an automated system for measuring feeding behavior of cattle. Behav. processes 148, 56–62. doi: 10.1016/j.beproc.2018.01.004 29330090

[B71] Sanches G. M. Duft D. G. Kölln O. T. Luciano A. C. D. S. De Castro S. G. Q. Okuno F. M.. (2018). The potential for RGB images obtained using unmanned aerial vehicle to assess and predict yield in sugarcane fields. Int. J. Remote Sens. 39, 5402–5414. doi: 10.1080/01431161.2018.1448484

[B72] Sarkar S. Cazenave A. B. Oakes J. McCall D. Thomason W. Abbot L.. (2020). High-throughput measurement of peanut canopy height using digital surface models. Plant Phenome J. 3, e20003. doi: 10.1002/ppj2.20003

[B73] Seabold S. Perktold J. (2010). “Statsmodels: Econometric and statistical modeling with python,” in Proceedings of the 9th Python in Science Conference, Vol. 57. 10–25080.

[B74] Settaluri V. S. Kandala C. V. K. Puppala N. Sundaram J. (2012). Peanuts and their nutritional aspects—a review. Food Nutr. Sci., 3–12.

[B75] Shahi T. B. Xu C. Y. Neupane A. Fleischfresser D. B. O’Connor D. J. Wright G. C.. (2023). Peanut yield prediction with UAV multispectral imagery using a cooperative machine learning approach. Electron. Res. Arch. 31, 3343–3361. doi: 10.3934/era.2023169

[B76] Shammi S. A. Meng Q. (2021). Use time series NDVI and EVI to develop dynamic crop growth metrics for yield modeling. Ecol. Indic. 121, 107124. doi: 10.1016/j.ecolind.2020.107124

[B77] Sharif M. Khan M. A. Iqbal Z. Azam M. F. Lali M. I. U. Javed M. Y. (2018). Detection and classification of citrus diseases in agriculture based on optimized weighted segmentation and feature selection. Comput. Electron. Agric. 150, 220–234. doi: 10.1016/j.compag.2018.04.023

[B78] Shi Y. Thomasson J. A. Murray S. C. Pugh N. A. Rooney W. L. Shafian S.. (2016). Unmanned aerial vehicles for high-throughput phenotyping and agronomic research. PloS One 11, e0159781. doi: 10.1371/journal.pone.0159781 27472222 PMC4966954

[B79] Soontranon N. Srestasathiern P. Rakwatin P. (2014). “Rice growing stage monitoring in small-scale region using ExG vegetation index,” in 2014 11th International Conference on Electrical Engineering/Electronics, Computer, Telecommunications And Information Technology (Ecti-Con). 1–5 (IEEE).

[B80] Sudduth K. A. Drummond S. T. Birrell S. J. Kitchen N. R. (1996). “Analysis of spatial factors influencing crop yield,” in Proceedings of the third international conference on precision agriculture, Madison, WI, USA. 129–139 (American Society of Agronomy, Crop Science Society of America, Soil Science Society of America).

[B81] Tao H. Xu S. Tian Y. Li Z. Ge Y. Zhang J.. (2022). Proximal and remote sensing in plant phenomics: Twenty years of progress, challenges and perspectives. Plant Commun., 100344. doi: 10.1016/j.xplc.2022.100344 35655429 PMC9700174

[B82] Thilakarathne N. Bakar M. S. Abas P. E. Yassin H. (2022). A cloud enabled crop recommendation platform for machine learning-driven precision farming. 22 (16), 6299. doi: 10.3390/s22166299 PMC9412477 36016060

[B83] Thorp K. R. Thompson A. L. Harders S. J. French A. N. Ward R. W. (2018). High-throughput phenotyping of crop water use efficiency via multispectral drone imagery and a daily soil water balance model. Remote Sens. 10, 1682. doi: 10.3390/rs10111682

[B84] Tunca E. Köksal E. S. Öztürk E. Akay H. Çetin Taner S. (2023). Accurate estimation of sorghum crop water content under different water stress levels using machine learning and hyperspectral data. Environ. Monit. Assess. 195, 877. doi: 10.1007/s10661-023-11536-8 37353582

[B85] Ubbens J. Cieslak M. Prusinkiewicz P. Parkin I. Ebersbach J. Stavness I. (2020). Latent space phenotyping: automatic image-based phenotyping for treatment studies. Plant Phenomics 2020. doi: 10.34133/2020/5801869 PMC7706325 33313558

[B86] Valentine H. (2016). The role of peanuts in global food security. Peanuts: Gene. Process. Util., 447–461. doi: 10.1016/B978-1-63067-038-2.00017-4

[B87] Van Impe J. F. Nicolaï B. M. Martens T. De Baerdemaeker J. Vandewalle J. (1992). Dynamic mathematical model to predict microbial growth and inactivation during food processing. Appl. Environ. Microbiol. 58, 2901–2909. doi: 10.1128/aem.58.9.2901-2909.1992 1444404 PMC183025

[B88] Van Rossum G. Drake J. F.L. (1995). Python reference manual (Centrum voor Wiskunde en Informatica Amsterdam).

[B89] Varela S. Pederson T. Bernacchi C. J. Leakey A. D. (2021). Understanding growth dynamics and yield prediction of sorghum using high temporal resolution UAV imagery time series and machine learning. Remote Sens. 13, 1763. doi: 10.3390/rs13091763

[B90] Variath M. T. Janila P. (2017). Economic and academic importance of peanut. Peanut Genome, 7–26. doi: 10.1007/978-3-319-63935-2_2

[B91] Virnodkar S. S. Pachghare V. K. Patil V. C. Jha S. K. (2020). Remote sensing and machine learning for crop water stress determination in various crops: a critical review. Precis. Agric. 21, 1121–1155. doi: 10.1007/s11119-020-09711-9

[B92] Virtanen P. Gommers R. Oliphant T. E. Haberland M. Reddy T. Cournapeau D.. (2020). SciPy 1.0: fundamental algorithms for scientific computing in Python. Nat. Methods 17, 261–272. doi: 10.1038/s41592-019-0686-2 32015543 PMC7056644

[B93] Weitkamp T. Karimi P. (2023). Evaluating the effect of training data size and composition on the accuracy of smallholder irrigated agriculture mapping in Mozambique using remote sensing and machine learning algorithms. Remote Sens. 15, 3017. doi: 10.3390/rs15123017

[B94] Williams E. J. Drexler J. S. (1981). A non-destructive method for determining peanut pod maturity. Peanut Sci. 8 (2), 134–141.

[B95] Yang W. Feng H. Zhang X. Zhang J. Doonan J. H. Batchelor W. D.. (2020). Crop phenomics and high-throughput phenotyping: past decades, current challenges, and future perspectives. Mol. Plant 13, 187–214. doi: 10.1016/j.molp.2020.01.008 31981735

[B96] Yoosefzadeh Najafabadi M. Hesami M. Eskandari M. (2023). Machine learning-assisted approaches in modernized plant breeding programs. Genes 14 (4), 777. 37107535 10.3390/genes14040777 PMC10137951

[B97] Young A. Mahan J. Dodge W. Payton P. (2020). BLOB-based AOMs: A method for the extraction of crop data from aerial images of cotton. Agriculture 10, 19. doi: 10.3390/agriculture10010019

[B98] Zeng L. Peng G. Meng R. Man J. Li W. Xu B.. (2021). Wheat yield prediction based on unmanned aerial vehicles-collected red–green–blue imagery. Remote Sens. 13, 2937. doi: 10.3390/rs13152937

[B99] Zerbato C. Rosalen D. L. Furlani C. E. A. Deghaid J. Voltarelli M. A. (2016). Agronomic characteristics associated with the normalized difference vegetation index (NDVI) in the peanut crop. Aust. J. Crop Sci. 10, 758–764. doi: 10.21475/ajcs.2016.10.05.p7167

[B100] Zheng C. Abd-Elrahman A. Whitaker V. Dalid C. (2022). Prediction of strawberry dry biomass from UAV multispectral imagery using multiple machine learning methods. Remote Sens. 14, 4511. doi: 10.3390/rs14184511

[B101] Zwietering M. H. Jongenburger I. Rombouts F. M. Van’t Riet K. (1990). Modeling of the bacterial growth curve. Appl. Environ. Microbiol. 56, 1875–1881. doi: 10.1128/aem.56.6.1875-1881.1990 16348228 PMC184525

